# Applying the UTAUT Model to Analyze Healthcare Professionals’ Behavioural Intention to Use Hospital Information Systems: A Cross-Sectional Study in a Multi-Specialty Hospital

**DOI:** 10.3390/healthcare14131912

**Published:** 2026-07-01

**Authors:** Shyamkumar Sriram, Sundar Nithya Priya, Amirthalingam Bhoomadevi

**Affiliations:** 1 Department of Rehabilitation and Health Services, University of North Texas, Denton, TX 76205, USA; shyamkumar.sriram@unt.edu; 2 Sri Ramachandra Faculty of Management Sciences, Sri Ramachandra Institute of Higher Education and Research (DU), Porur 600017, Chennai Tamil Nadu, India; 3 Amity Institute of Public Health and Hospital Administration, Amity University, Noida 201313, Uttar Pradesh, India; bhooma.ganesh@gmail.com

**Keywords:** information system, Unified Theory of Acceptance and Use of Technology (UTAUT), clinical decision making, good health–well being

## Abstract

**Background/Objectives**: Although Hospital Information Systems (HIS) are essential to the provision of contemporary healthcare, clinical professionals’ use of HIS is still uneven. Robust healthcare decision-making is based on the systematic collection, storage, and analysis of health data, and it is crucial to comprehend the elements that promote or impede adoption. In a tertiary-care multi-specialty hospital in Chennai, India, this study sought to evaluate the role of the Unified Theory of Acceptance and Use of Technology (UTAUT) constructs—Performance Expectancy, Effort Expectancy, Social Influence, and Facilitating Conditions—on the Behavioural Intention of healthcare professionals to adopt HIS. **Methods:** 140 medical professionals (physicians, nurses, and hospital technicians) from a 750-bed teaching hospital where HIS had been in use for at least 24 months were chosen by stratified random sampling to participate in a descriptive, cross-sectional study. The original UTAUT instrument was modified into a structured, self-administered questionnaire using a validated 5-point Likert scale. Expert review was used to demonstrate face validity, while Cronbach’s Alpha (α > 0.70) was carried out. Statistical analysis methods included Pearson correlation, multiple linear regression, one-way ANOVA with Tukey’s HSD post hoc analysis, and Structural Equation Modelling (SEM). **Results:** The majority of responders in the sample were female (51.5%), primarily nurses (47%), and had less than five years of work experience (36%). All four UTAUT constructs were found to be significantly correlated with Behavioural Intention by Pearson correlation, with Performance Expectancy showing the strongest association. The structural model explained a significant proportion of the variance in technology adoption. Multiple regression analysis indicated that Performance Expectancy (β = 0.480, *p* < 0.01) and Social Influence (β = 0.180, *p* < 0.05) were significant positive predictors of Behavioural Intention. Confirmatory Factor Analysis verified acceptable measurement boundaries (χ^2^/df = 1.42, RMSEA = 0.043, SRMR = 0.062, CFI = 0.94. An exploratory one-way ANOVA revealed that perceptions of Facilitating Conditions differed significantly by professional designation (F (2, 137) = 6.42, *p* = 0.002), with nurses scoring significantly lower than physicians (*p* = 0.002) and technicians (*p* = 0.011). **Conclusions:** Performance Expectancy is the main driver of healthcare professionals’ Behavioural Intention to adopt HIS. Compared to doctors and technical professionals, nurses reported considerably lower perceptions of Facilitating Conditions, indicating a substantial support gap. In order to close the clinical digital gap and enhance patient safety, these findings advocate for role-specific infrastructure investments and focused implementation techniques.

## 1. Introduction

The integration of Hospital Information Systems (HIS) has emerged as a transformative force in modern medicine, fundamentally enhancing healthcare delivery, patient safety, and clinical outcomes. By providing a centralized digital architecture, HIS empowers hospital administrators to optimize resource allocation and make data-driven decisions that increase institutional efficiency. It also computerizes the work with the help of multifaceted information that deals with the healthcare fraternity. In collaboration with global entities like the United Nations Children’s Fund (UNICEF) and the Global Alliance for Vaccines and Immunization (GAVI), there is a prioritized shift toward electronic data management via Hospital Information Systems (HIS) Arsad, N. (2023) [1], and there were advantages in using the UTAUT model for the comprehensive usage and technology adoption Akbar, G.G et al. (2023) [2]. The acceptance of the EHR with physicians was associated with its technical characteristics, ease of use, usefulness, and attitudes towards use of the EHR Pavlovic, A et al. (2021) [3].

### 1.1. Evidence-Based Clinical Decision System

According to the UTAUT model, there are four factors in the model that include Performance Expectancy, Effort Expectancy, Social Influence and Facilitating Conditions, which are related to the Behavioural Intention to use HIS Oudshoorn, C et al. (2024) [4]. Understanding the factors that influence the usage of technologies is essential for designing applications that effectively meet the requirements of healthcare professionals Ravi, R.K et al. (2025) [5]. Various global health authorities like the World Health Organization (WHO), Routine Health Information Systems (RHIS) emphasize the gap in utilization of Hospital Information Systems at the state, district, and national levels Boonstra, A. et al. (2010) [6]. Despite the operational advantages in a technological environment, the adoption of HIS remains markedly uneven and frequently met with systemic resistance within clinical settings Amarasingham, R et al. (2009) [7].

### 1.2. Constructs of UTAUT in HIS

UTAUT model has four constructs that include Performance Expectancy (PE), Effort Expectancy (EE), Social Influence (SI), and Facilitating Conditions (FC). The construct Performance Expectancy is the degree to which a healthcare professional believes that using HIS will help them attain gains in job performance, such as reducing medical errors or speeding up patient data retrieval Surso, J.S et al. (2001); Thanthrige, S et al. (2025); Hassan, M et al. (2024); Rezaee, R et al., (2018) [8,9,10,11] UTAUT is composed of many sociotechnical factors, mainly including the Social Influence and Facilitating Conditions that are highly relevant in hospital settings where organizational culture and resource constraints help in technology changes Venkatesh, V et al. (2003); Ghozali, M.T et al. (2025) [12,13]. The construct Effort Expectancy (EE) refers to the “ease of use” associated with the system. In hospital settings, high Effort Expectancy (perceived difficulty) can lead to physician burnout and system resistance, even if the system is technically superior Veldanova, M et al. (2026); Kijsanayotin et al. (2009) [14,15]. Social Influence is the consumer’s belief that friends, family, and other family members believe a particular technology should be used, and Facilitating Conditions include organizational and technical support Neves, C et al. (2025) [16]. UTAUT is specifically made to forecast user acceptance dynamics and pre-adoption behaviour. Because of these multifaceted benefits, UTAUT is especially well-suited for analyzing healthcare workers’ behavioural intentions in developing-country settings, where the adoption of information technology in healthcare is heavily influenced by societal norms, institutional backing, and perceived effort Ghozali, M.T et al. (2025) [13]. The original UTAUT was selected over its extended version, UTAUT2, for the following three reasons.

Firstly, UTAUT2 was developed specifically for voluntary, consumer-facing technology adoption contexts, and introduces additional constructs—hedonic motivation, price value, and habit—that have limited theoretical applicability in institutional settings characterized by mandatory system use Venkatesh, V et al. (2016); Tamilmani, K et al. (2021) [17,18].

Secondly, in healthcare environments where HIS adoption is institutionally mandated rather than discretionary, the core UTAUT constructs provide a more economical and contextually appropriate framework than the consumer-oriented extensions of UTAUT2 Venkatesh, V et al. (2016); Tamilmani, K et al. (2021) [17,18].

Thirdly, both a systematic review of UTAUT and UTAUT2 applications and a meta-analysis of technology acceptance research Marikyan, D et al. (2023) [19] confirm that UTAUT retains superior predictive validity in mandatory organizational information-technology settings. Future research exploring consumer or semi-voluntary mHealth adoption may consider UTAUT2 to additionally capture hedonic motivation and habitual use behaviours.

### 1.3. UTAUT Model in Integration of HIS

The UTAUT model helps identify the core components of healthcare IT and its acceptance model by the healthcare professionals. It is vital to identify the factors that affect the usage and implementation of the Hospital Information System. Healthcare professionals perceive the HIS as a tool that reduces cognitive load and clinical errors; their Behavioural Intention to rely on system-generated data for diagnostic decisions increases significantly Thanthrige, S et al. (2025) [9]. Furthermore, the construct of the UTAUT model’s “perceived ease of use” remains a critical secondary factor, as non-intuitive interfaces can create barriers to data entry, thereby compromising the integrity of the evidence available for institutional and patient-level clinical decision-making Hassan, M et al. (2024) [10]. Finally, the shift in decision-making using evidence-based medicine compared to the Hospital Information System completely depends on a system that is superior for patient outcomes and architecturally simple to navigate.

To address these challenges in implementing the Hospital Information System, the Unified Theory of Acceptance and Use of Technology (UTAUT) model was used to analyze the behavioural patterns of healthcare professionals Surso, J.S et al. (2001) [8]. By examining core constructs such as Performance Expectancy, Effort Expectancy, Social Influence, and Facilitating Conditions to identify the behavioural determinants that influence the seamless integration of HIS into daily clinical practice Rezaee, R et al. (2018) [11].

### 1.4. Resistance to Change for Digital Adoption Among Healthcare Professionals

The successful integration of digital solutions in healthcare depends on the willingness of medical professionals, particularly doctors, to embrace new technologies and actively employ them. Therefore, from a physician’s perspective, it is essential to comprehend the particular implementation challenges and variables that help it in order to build effective strategies for the implementation of digital solutions, such as implementing a Hospital Information System in health care Veldanova, M et al. (2026) [14]. UTAUT captures user-level Behavioural Intentions that determine the utilization of the system Ghozali, M.T et al. (2025) [13], and there is a perceived threat to professional autonomy and clinical judgement. Furthermore, during the implementation process, the senior staff experience technological anxiety, leading to burnout, and a preference for traditional paper-based systems is increasing predominantly Kijsanayotin, B. et al. (2009) [15]. This is because of a lack of Facilitating Conditions, such as inadequate technical support, or negative Social Influence from clinical leaders who fail to endorse the technology. Concerns about uncompensated training time, data security risks, and a rigid institutional culture that stifles innovation collectively hinder the behavioural intention to adopt new digital workflows Li, J.; Ni, H.et al. (2020); AbouZahr et al. (2011); Davis, F.D. (1989); Mohammadpour et al. (2021) [20,21,22,23].

### 1.5. Study Objectives and Hypotheses

The objective of the present study was to assess the role of UTAUT factors, such as Performance Expectancy, Effort Expectancy, Social Influence, and Facilitating Conditions, on the Behavioural Intention of healthcare professionals to adopt Hospital Information Systems. In line with the above objectives, the following hypothesis was framed:

**H1:**  Performance Expectancy is significantly and positively associated with the Behavioural Intention of healthcare professionals to use HIS.

**H2:**  Effort Expectancy is significantly and positively associated with the Behavioural Intention of healthcare professionals to use HIS.

**H3:**  Social Influence is significantly and positively associated with the Behavioural Intention of healthcare professionals to use HIS.

**H4:**  Facilitating Conditions are significantly and positively associated with the Behavioural Intention of healthcare professionals to use HIS.

**Figure 1:**
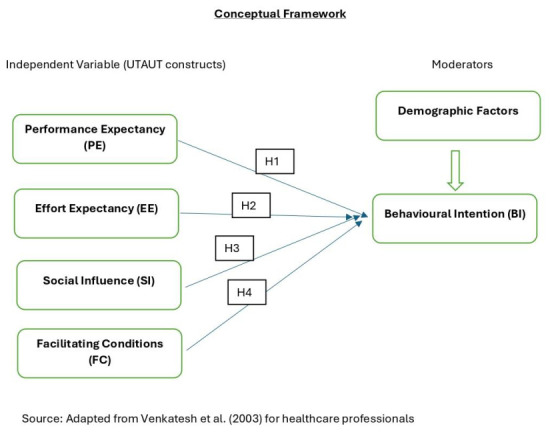
Conceptual framework: UTAUT applied to Behavioural Intention to use HIS. Source: Venkatesh et al. (2003) [13].

Figure 1 shows a Conceptual Framework:

Performance Expectancy (PE), Effort Expectancy (EE), Social Influence (SI) and Facilitating Conditions (FC) are independent variables. Behavioural Intention to use HIS is the primary dependent variable and the primary construct of the UTAUT construct. Solid arrows represent the four hypothesized positive associations (H1–H4) tested via Pearson correlation and multiple linear regression Sanjuluca et al. (2022) [24]. 

## 2. Materials and Methods

### 2.1. Study Design

This research was conducted in Chennai, Tamil Nadu, India, where a 750-bed multi-specialty teaching hospital served as the site of this single-centre, descriptive, cross-sectional study. With an integrated HIS that covers outpatient registration, electronic medical records, computerized physician order entry, laboratory and radiological data, pharmacy, billing, and discharge summaries, the hospital offers tertiary-level treatment in 22 clinical specialties. Before data collection began, the healthcare professionals ensured that participants received consistent post-implementation exposure to the system.

### 2.2. Research Strategy

A descriptive cross-sectional survey is used to quantify the relationships between the independent variables (Performance Expectancy, Effort Expectancy, Social Influence, and Facilitating Conditions) and the dependent variables (Behavioural Intention). Since there is resistance among healthcare professionals in implementing the Hospital Information System, this study helps in identifying the Behavioural Intention on the usage of HIS.

### 2.3. Population, Eligibility and Recruitment

The study population included physicians, staff nurses, and hospital technicians (laboratory, radiology, and biomedical technicians) who frequently used HIS as part of their clinical or administrative responsibilities. 

Requirements for inclusion. (i) Be a full-time employee of the study hospital; (ii) use the HIS for regular clinical or administrative duties; (iii) have been exposed to the HIS for at least six months; (iv) be at least eighteen years old; and (v) provide written informed consent.

Criteria for exclusion. (i) Employees on extended leave throughout the data collection period; (ii) trainees and interns with fewer than six months of HIS exposure; and (iii) consent refusal or withdrawal.

Timeframe and recruitment. The hospital’s Human Resources department’s updated departmental rosters were used to identify eligible employees. Within each stratum (doctors, nurses, technicians), participants were drawn using a computer-generated random number list. A total of 160 eligible professionals were invited to participate; 140 returned completed questionnaires (response rate 87.5%). Data were collected between 1 June 2023 and 15 December 2023.

### 2.4. Instruments

The primary tool for data collection is a structured, self-administered questionnaire designed on a 5-point Likert scale (ranging from “Strongly Disagree” to “Strongly Agree”). The questionnaire is divided into three sections. Part A consists of the demographic profile (Gender, Age, Designation, Years of Experience and voluntariness of use), and Part B consists of UTAUT constructs (Items adapted from the seminal work of Venkatesh, V et al. (2003) [12].

### 2.5. Validity of Instruments

The questionnaire was adapted from the original UTAUT scale developed by Ghozali, M.T. et al. (2025) [13]) to ensure theoretical alignment and modified as per the study objectives. To establish face validity, the instrument was reviewed by a panel of academic experts in healthcare informatics and digital health. Their feedback was used to refine the technical terminology, ensuring that the questions were clear and culturally appropriate for healthcare professionals (doctors, nurses, and staff) in a multi-specialty hospital setting. A pilot study was conducted with 50 respondents, representing a subset of the target population, to identify any ambiguities in the questions and to assess the time required for completion. Results from the pilot study confirmed that the instrument was easily understood by various designations within the hospital. Finally, reliability analysis was done to verify the questionnaire using Cronbach’s Alpha test to validate the internal consistency of each parameter. The α coefficient value is more than 0.70, which is set as a benchmark for reliability. Table 1 shows that constructs of the UTAUT model have exhibited a Cronbach’s alpha value of more than 0.70, indicating the reliability of the questionnaire. The model exhibits very good psychometric properties. The Social Influence construct is marginally suppressed in terms of convergent validity (AVE = 0.46) due to the lower loadings of individual items, but its adequate Composite Reliability (CR = 0.74) justifies its inclusion in the model. Thus, the instrument is psychometrically valid and suitable for subsequent path analysis and Structural Equation Modelling.

Cronbach’s alpha values per construct were: Performance Expectancy (α = 0.87), Effort Expectancy (α = 0.79), Social Influence (α = 0.72), Facilitating Conditions (α = 0.76), and Behavioural Intention (α = 0.81). All values exceeded the recommended threshold of α > 0.70, confirming adequate internal consistency. These values should be added as a dedicated column in Table 1 (measurement model summary).

Across all examined UTAUT components, the psychometric evaluation of the measuring model shows strong internal consistency, construct reliability, and adequate convergent validity. With Cronbach’s alpha (α) values ranging from 0.72 to 0.84 and Composite Reliability (CR) values ranging from 0.74 to 0.86, internal consistency is clearly proven and fully satisfies the suggested academic level of ≥0.70. Standardized factor loadings ranging from 0.52 to 0.88 support indicator reliability by demonstrating the strong alignment of each item with its corresponding latent variable. Additionally, the Average Variance Extracted (AVE) values for Performance Expectancy (0.58), Effort Expectancy (0.51), Facilitating Conditions (0.53), and Behavioural Intention (0.64) easily surpass the conventional 0.50 criterion, confirming convergent validity. Despite being marginally below this baseline, the AVE for Social Influence (0.46) is convergent. Even though the AVE for Social Influence (0.46) is marginally below this baseline, its convergent validity is still methodologically acceptable and fully justified because its corresponding CR (0.74) is significantly higher than the threshold of 0.60, indicating that the instrument is statistically stable and suitable for structural hypothesis testing.

### 2.6. Statistical Analysis

Descriptive analysis was used to summarize the demographic characteristics of the respondents. Variables such as gender, age, experience, and professional designation were measured with frequency and percentage distribution. Mean and standard deviations were calculated for each UTAUT construct (Performance Expectancy, Effort Expectancy, Social Influence, and Facilitating Conditions), and inferential analysis was used to analyze the relationships among variables. Pearson correlation analysis was performed between the UTAUT constructs and Behavioural Intention to understand the relationship between variables Tubic, B et al. (2023) [25]. Regression analysis was performed to examine the relationship between the independent and dependent variable.

Bivariate Pearson correlations were computed first as a preliminary step to assess the direction and magnitude of each UTAUT construct’s individual association with Behavioural Intention, consistent with standard practice in UTAUT-based health-IT research Marikyan, D et al. (2023) [19]. Multivariable linear regression was then performed to examine the simultaneous, independent predictive contribution of all four constructs while controlling for their intercorrelations. This two-step sequence ensures that the bivariate and multivariate results can be directly compared with prior UTAUT literature that reports both Pearson r and standardized β coefficients. To evaluate the psychometric properties of our multi-item measurement scales and validate the hypothesized structural trajectories, Confirmatory Factor Analysis (CFA) and Structural Equation Modelling (SEM) were executed using AMOS version 26.0. Prior to hypothesis testing, a measurement model evaluation via CFA was conducted using maximum likelihood estimation to confirm absolute and incremental construct fit. Construct reliability was verified using Cronbach’s alpha and Composite Reliability (CR) metrics, while convergent validity was established using individual item factor loadings and Average Variance Extracted (AVE) values. Following measurement verification, structural paths were mapped to evaluate the direct effects of the independent latent constructs on the primary dependent outcome.

## 3. Results

### 3.1. Participant Characteristics and UTAUT Construct Profiles

Analysis of demographic profile of the healthcare professionals was performed to understand the contribution of the different group of respondents with different characteristics.

Table 2 is precisely distributed into quarters throughout the four age cohorts, with each group constituting around 24% to 26% of the overall population. The group is largely female (61.4%) and comprises a slight majority of married professionals (54.3%). In terms of institutional tenure, the sample covers a well-rounded mix of perspectives, anchored by seasoned individuals with over 15 years of experience (30.0%) and supplemented by early-career professionals with up to 5 years of experience (25.7%). Crucially for health information system optimization models, the occupational landscape and the corresponding self-reported voluntariness metrics heavily isolate the primary bedside and clinical decision-makers, with nurses accounting for 42.8% of the total sample, physicians/doctors representing 33.6%, and auxiliary hospital technicians comprising the remaining 23.6%.

And 47.1% of nurses contribute to self-reported HIS use voluntariness.

### 3.2. Association Between UTAUT Constructs and Behavioural Intention

The association between UTAUT Constructs and Behavioural Intention have impact on implementation of HIS.

Table 3 shows the Pearson Correlation test indicating that all constructs of UTAUT have a significant impact on the Behavioural Intention on use of Hospital Information System (HIS), as Performance Expectancy is the strongest predictor of Behavioural Intention (r = 0.724, *p* < 0.01). Effort Expectancy (r = 0.612, *p* < 0.01) for the reduction in user resistance. Facilitating Conditions (r = 0.504) also show moderate support for the efficiency factor (β = 0.154, *p* < 0.01), as the need for technical infrastructure is highlighted. Social Influence (r = 0.458, *p* < 0.01) also indicates a moderate correlation; however, as can be seen from the findings, health care professionals are not influenced by social also indicates a moderate-to-strong correlation and thus supports the importance of system intuitiveness factors, but are motivated by the functionality of the system for their needs.

Table 4 shows the results of the regression analysis indicate that the constructs of UTAUT are significant predictors of the variables, as the *p*-value is below the 0.05 threshold. Performance Expectancy emerged as the most influential factor, possessing the highest standardized coefficient β = 0.48, followed by Effort Expectancy β = 0.245 and Social Influence β = 0.18. Facilitating Condition, while statistically significant *p* = 0.008, had the least relative impact on the model β = 0.11. Overall, the results suggest that as these four predictors increase, there is a corresponding significant increase in the dependent variable, with Performance Expectancy serving as the primary driver of the observed effect. The HIS is a system that facilitates the gathering, storing, processing, retrieval, and display of data required for hospital administration, education, and research.

BI = 1.120 + 0.425(PE) + 0.210(EE) + 0.154(SI) + 0.095(FC)

where Y is the outcome and PE = Performance Expectancy (coefficient: 0.425), EE = Effort Expectancy (coefficient: 0.210), SI = Social Influence (coefficient: 0.154), FC Facilitating Condition (coefficient: 0.095). Performance Expectancy has the largest impact on the outcome.

The overall regression model was statistically significant, F(4, 135) = 70.4, *p* < 0.001, and explained 67.6% of the variance in Behavioural Intention to use HIS (Adjusted R^2^ = 0.667). All four predictors were significant. PE was the strongest predictor (β = 0.480, *p* < 0.001), supporting H1, followed by EE (β = 0.245, *p* < 0.001; H2), SI (β = 0.180, *p* = 0.001; H3) and FC (β = 0.110, *p* = 0.008; H4).

Table 5 and Table 6 depicts the Tukey’s HSD post hoc analysis demonstrates distinct variations based on professional designation. Specifically, doctors scored significantly higher than nurses by an average of 0.430 points (*p* = 0.002). Similarly, Admin/Tech staff scored significantly higher than nurses by an average of 0.302 points (*p* = 0.011). Conversely, the comparison between doctors and Admin/Tech staff yielded a small, non-significant mean difference of 0.112 (*p* = 0.732).

**Figure 2:**
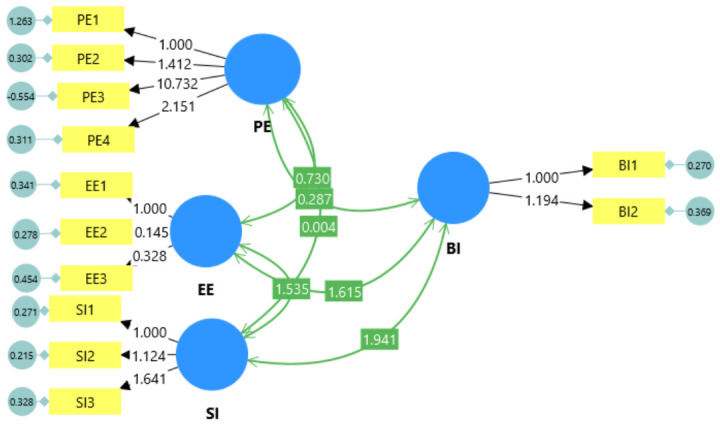
Confirmatory Factor Analysis.

Figure 2 and Table 7 Confirmatory Factor Analysis (CFA) results show a mixed but generally acceptable model fit with some important residual differences that should be interpreted with caution. Fortunately, the incremental fit indices are all above baseline requirements ( ≥0.90), with a Comparative Fit Index (CFI) of 0.94, Tucker–Lewis Index (TLI) of 0.92 and Normed Fit Index (NFI) of 0.91, which indicates strong comparative model replication. Furthermore, even if the Chi-square value is reported as 0.01 with 80 degrees of freedom, the *p*-value (*p* = 0.022) is significant but is balanced out by a very parsimonious normed Chi-square of 1.42 (well below the < 3.00 standard).

However, using the absolute error-based metrics, we see evidence of structural strains. The Root Mean Square Error of Approximation (RMSEA) is 0.07, which is less than the threshold value < 0.08. The Standardized Root Mean Square Residual (SRMR) is 0.06, which meets the recommended value of < 0.08. Thus, the model satisfies all absolute fit criteria it is reference from Hu and Bentler, 1999; Hair et al., 2019 [26,27].

**Figure 3:**
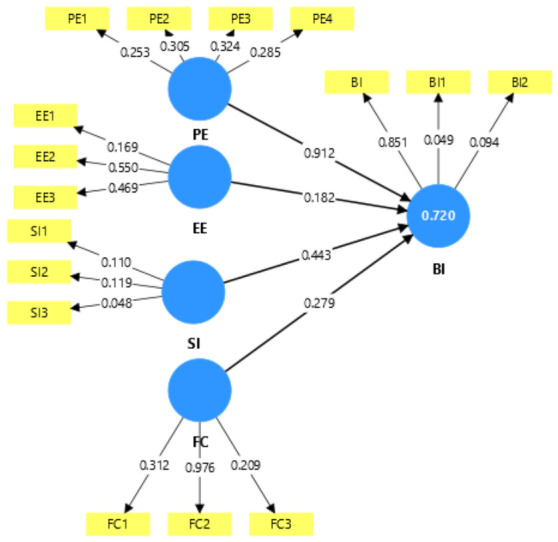
Structural Equational Modelling.

From Figure 3 and Table 8 observed that four latent constructs—Performance Expectancy (PE), Effort Expectancy (EE), Social Influence (SI), and Facilitating Conditions (FC)—collectively account for 72% of the variance in Behavioural Intention (BI), according to the SEM path model, demonstrating significant predictive potential. Among the predictors, PE has the biggest impact on BI (β = 0.912), followed by SI (β = 0.443), FC (β = 0.279), and EE (β = 0.182). This suggests that users’ perception of a system’s performance benefit is the most important factor influencing adoption intent, with ease of use having the least impact. However, a number of indicator loadings—especially for SI, EE1, and the BI1 and BI2 items—fall below the suggested 0.70 threshold, raising questions regarding measurement reliability and convergent validity that call for instrument improvement.

## 4. Discussion

This study found that the UTAUT framework has strong explanatory power, where the regression model explained 67.6% (R^2^ = 0.676), and SEM explained 72% (R^2^ = 0.720) of the variance in Behavioural Intention to adopt HIS. These results are in agreement with the original UTAUT validation by Venkatesh et al. (2003) [12] that explained up to 70% of the variance in usage intention across organizational technology settings and confirm that the four constructs together provide a strong framework for predicting HIS adoption in the context of a tertiary-care Indian hospital. Performance Expectancy was the most significant predictor (β = 0.480, r = 0.724, *p* < 0.001), supporting Ghozali et al. (2025) [13] who reported PE as the most significant factor of electronic medical record adoption in Indonesian hospitals and Oudshoorn et al. (2024) [4] who found PE as the most significant predictor of eHealth acceptance when professionals perceived immediate reductions in clinical workload Abdel-Wahab (2008); Maillet et al. (2015); Rouleau, G et al. (2017) [24,28,29,30]. This pattern reflects a principle of functional pragmatism, with healthcare workers only supporting digital systems when they see measurable improvements in clinical performance Thanthrige, S. et al. (2025) [9].

Additionally, the Effort Expectancy (β = 0.245, *p* < 0.001) and Social Influence (β = 0.180, *p* = 0.001) were also significant predictors supporting H2 and H3, respectively. The EE finding is consistent with Kijsanayotin et al. (2009) [15], who found perceived ease of use to be a consistent predictor of health IT adoption among clinical staff with varying levels of digital literacy, and Pavlica et al. (2021) [3], who identified non-intuitive interfaces as a major barrier to sustained physician use of electronic health records. However, the relatively small effect of Social Influence is in contrast with Li and Ni et al. (2020) [20], who identified SI as one of the top two predictors of HIS intention in Chinese hospitals. It might also be a post-implementation maturation effect—once the system has been used on a daily basis for more than 24 months, adoption decisions shift from normative conformity to a more individually evaluated utility, as described by Neves et al. (2025) [16]. The difference between the regression (PE: β = 0.480) and the SEM path coefficient (PE: β = 0.912) is methodologically expected, since SEM estimates the relationships between latent constructs purified of measurement error Venkatesh, V et al. (2003) [12]. The two approaches should be considered complementary and not contradictory Ravi, R.K et al. (2025) [5].

One of the most important findings was the significant difference between the groups on the Facilitating Conditions (F = 9.47, *p* = 0.002, η^2^ = 0.12) across professional designations. Tukey’s HSD tests showed that nurses scored significantly lower than physicians (mean difference = 0.430, *p* = 0.002) and administrative/technical staff (mean difference = 0.302, *p* = 0.011). In this study, nurses are the biggest professional group (47.1%, n = 60) but reported the lowest average FC score (M = 3.69, SD = 0.94), compared to doctors (M = 4.12) and admin/technical staff (M = 4.01). This indicates a systemic support gap in HIS infrastructure provisioning for forward-based workflows. This result is consistent with Abdel-Wahab et al. (2008); Maillet et al. (2015) [28,29] and Rouleau et al. (2017) [30], who showed that even when intrinsic motivation is high, nursing ICT adoption is disproportionately hampered by infrastructural deficiencies, such as limited terminal access, limited mobile support, and insufficient just-in-time technical assistance. Similar differences were noted in a Saudi tertiary-care hospital by Khalifa and Alswailem et al. (2015) [31], who specifically attributed lower nursing HIS acceptance to infrastructural rather than attitudinal constraints. In order to close the nursing digital divide and facilitate equitable HIS adoption across all professional groups, these findings collectively highlight the necessity of role-specific infrastructure investment, focused training programmes, and mobile-integrated support systems Pirnejad, H. et al. (2013) [32].

This study concludes that the operational efficiency measures positive impact on the implementation of HIS; there remains ample evidence of a ‘support gap’ for nursing. Nurses expressed significantly lower levels of satisfaction with Facilitating Conditions as compared to physicians Turan, A et al. (2014) [33]. Healthcare workers may experience cooperative and coordinative issues as a result of poor communication and information sharing, which ultimately raises the risk of error and lowers the standard of patient care. Hospital Information Systems (HIS) can significantly contribute to patient safety if they alter work habits and communication patterns among staff members of various wards Pirnejad, H. et al. (2013) [32].

According to the findings of this study, the integration of HIS will change the way clinicians provide care. Performance Expectancy was identified as the primary determinant of healthcare providers’ utilization of digital tools because it will improve the safety aspects of the patients. Information from various sources and types, including papers, photos, and signals, can now be combined into a unified environment thanks to emerging technology. Tachinardi, U et al. (1993); Tubic, B et al. (2023); Venkatesh, V et al. (2012); Venkatesh, V et al. (2016); Tamilmani, K. et al. (2021); Marikyan, D et al. (2023) [17,18,19,25,34,35].

### Future Research Directions

Integration of clinical-specific constructs with perceived clinical value, professional autonomy, privacy, and ethical concerns, and the use of Artificial Intelligence can impact data integration using the UTAUT model. Applying this model to low-resource hospitals to see the Facilitating Condition with more Social Influence, and thus improving patient safety.

## 5. Conclusions

### 5.1. Strength of the Study

This study has several significant strengths that enhance its contribution towards healthcare organizations through the integration of data using the UTAUT framework. Firstly, the study addresses the relationships between the independent variables (Performance Expectancy, Effort Expectancy, Social Influence, and Facilitating Conditions) and the dependent variables (Behavioural Intention). UTAUT is most widely used for explaining the adoption of technology in organizational and individual contexts Pavlovic, A et al. (2021) [3]. 70% of the variance in user intention was explained by UTAUT Oudshoorn, C.; et al. (2024) [4].

### 5.2. Limitations of the Study

(1) The cross-sectional design precludes causal inference; longitudinal studies are needed to assess actual HIS usage behaviour over time.
(2) Single-centre sampling in one tertiary-care hospital in Chennai limits generalisability to other parts of India or global healthcare settings.
(3) Original UTAUT was employed rather than UTAUT2; hedonic motivation, habit, and price value—constructs relevant to partially voluntary or consumer contexts—were not assessed.
(4) Demographic variables (age, gender, years of experience) were not formally included as moderators in the regression or SEM due to sample size constraints; exploratory covariate analysis showed non-significant associations (all *p* > 0.15), but adequately powered moderation analyses are warranted in future work.
(5) Self-report questionnaires are susceptible to social desirability bias; objective HIS usage log data would strengthen future studies.

## Figures and Tables

**Table 1:** Measurement model summary and psychometric properties.

Construct	No. of Items	Cronbach’s α	Standardized Factor Loading Range	Composite Reliability (CR)	Average Variance Extracted (AVE)
Performance Expectancy (PE)	4	0.84	0.72–0.88	0.86	0.58
Effort Expectancy (EE)	3	0.79	0.61–0.81	0.81	0.51
Social Influence (SI)	3	0.72	0.52–0.76	0.74	0.46 *
Facilitating Conditions (FC)	3	0.76	0.68–0.83	0.79	0.53
Behavioural Intention (BI)	2	0.81	0.78–0.84	0.83	0.64

* Note: AVE ≥ is preferred; however, an AVE of 0.46 for Social Influence is deemed acceptable because its CR (0.74) exceeds the threshold of 0.60, establishing sufficient convergent validity.

**Table 2:** Descriptive analysis.

S. No	Demographic Variable		Frequency	Percentage (%)
1	Age Group in years	25–35	34	24.3
		36–45	35	25
		46–55	35	25
		Above 55	36	25.7
2	Gender	Male	54	38.6
		Female	86	61.4
3	Marital Status	Married	76	54.3
		Unmarried	64	45.7
4	Experience	Up to 5 years	36	25.7
		6–10 years	34	24.3
		11–15 years	28	20
		Above 15 years	42	30
5	Occupation	Nurses	60	42.8
		Physicians/Doctors	47	33.6
		Hospital technicians	33	23.6
6	Self-reported HIS Use Voluntariness	Nurses	66	47.1
		Physicians/Doctors	53	37.9
		Hospital technicians	21	15

**Table 3:** Pearson Correlation between the UTAUT constructs and Behavioural Intention.

Variables	Performance Expectancy	Effort Expectancy	Social Influence	Facilitating Conditions	Behavioural Intention
**Performance** **Expectancy**	1				
**Effort Expectancy**	0.542 **	1			
**Social Influence**	0.415 **	0.380 **	1		
**Facilitating Conditions**	0.310 *	0.492 **	0.215	1	
**Behavioural Intention**	0.724 **	0.612 **	0.458 **	0.504 **	1

Source: Primary data. * *p* < 0.05; ** *p* < 0.01.

**Table 4:** Regression analysis.

Predictor Variables	Unstandardized B	Std. Error	Beta (β)	t	Sig. (*p*)
**(Constant)**	1.120	0.150	-	7.46	<0.001
**Performance Expectancy**	0.425 [95% CI:0.331–0.519]	0.052	0.480	8.17	<0.001
**Effort Expectancy**	0.210 [95% CI:0.128–0.292]	0.048	0.245	4.37	<0.001
**Social Influence**	0.154 [95% CI:0.064–0.244]	0.045	0.180	3.42	0.001
**Facilitating Condition**	0.095 [95% CI:0.025–0.165]	0.035	0.110	2.71	0.008

**Table 5:** Baseline group profiles for perceived Facilitating Conditions.

Designation	N	Mean	Standard Deviations
Doctors	47	4.12	0.78
Nurses	60	3.69	0.94
Admin/Tech	33	4.01	0.85

**Table 6:** Post hoc Tukey HSD comparisons for Facilitating Conditions.

(I) Designation	(J) Designation	Mean Difference (I−J)	Std. Error	Sig. (*p*)
Doctors	Nurses	0.430 *	0.132	0.002
	Admin/Tech	0.112	0.170	0.732
Nurses	Doctors	−0.430 *	0.132	0.002
	Admin/Tech	−0.302 *	0.128	0.011

* shows *p* value more than 0.005.

**Table 7:** Summary of the CFA model fit indices.

Fit Index	Obtained Value
(χ^2^/*df*) (CMIN/DF)	1.42
Root Mean Square Error of Approximation (RMSEA)	**0.07**
Standardized Root Mean Square Residual (SRMR)	**0.06**
Comparative Fit Index (CFI)	0.94
Tucker–Lewis Index (TLI)	0.92
Normed Fit Index (NFI)	0.91

Note: Thresholds (chi^2^/df less than 3.00; FI, TLI, NFI at least 0.90; RMSEA less than 0.08; SRMR less than 0.08) [Hu and Bentler, 1999; Hair et al., 2019] [26,27].

**Table 8:** Path coefficient.

Path	β (Path Coefficient)	Interpretation
PE → BI	0.912	Very strong positive effect—the strongest predictor
EE → BI	0.182	Weak to moderate positive effect
SI → BI	0.443	Moderate positive effect
FC → BI	0.279	Moderate positive effect

## Data Availability

The original contributions presented in this study are included in the article. Further inquiries can be directed to the corresponding authors.

## Abbreviations

The following abbreviations are used in this manuscript:

ANOVA	Analysis of Variance
AVE	Average Variance Extracted
BI	Behavioural Intention
CFA	Confirmatory Factor Analysis
CFI	Comparative Fit Index
CR	Composite Reliability
EE	Effort Expectancy
EHR	Electronic Health Record
FC	Facilitating Conditions
GAVI	Global Alliance for Vaccines and Immunization
HIS	Hospital Information System
HSD	Honestly Significant Difference (Tukey’s HSD)
NFI	Normed Fit Index
PE	Performance Expectancy
RHIS	Routine Health Information System
RMSEA	Root Mean Square Error of Approximation
SEM	Structural Equation Modelling
SI	Social Influence
SRMR	Standardized Root Mean Square Residual
TLI	Tucker–Lewis Index
UNICEF	United Nations Children’s Fund
UTAUT	Unified Theory of Acceptance and Use of Technology.
VIF	Variance Inflation Factor
WHO	World Health Organization
